# A cochlea-sparing strategy for non-invasive control of intracranial schwannomas via peripheral irradiation and anti-PD-1 therapy enhanced by STING activation

**DOI:** 10.7150/thno.123726

**Published:** 2026-01-21

**Authors:** Zhenzhen Yin, Simeng Lu, Limeng Wu, Yao Sun, Day Caven Blake, Jie Chen, Lukas D. Landegger, William Ho, Bingyu Xiu, Adam P. Jones, Alona Muzikansky, Helen A. Shih, Konstantina M. Stankovic, Scott R. Plotkin, Lei Xu

**Affiliations:** 1 Edwin L. Steele Laboratories, Department of Radiation Oncology, Massachusetts General Hospital, Harvard Medical School, Boston, MA, 02114, USA.; 2 Department of Otolaryngology - Head and Neck Surgery and Department of Neurosurgery, Stanford University School of Medicine, Stanford, CA, 94305, USA.; 3 Biostatistics Center, Massachusetts General Hospital, Harvard Medical School, Boston, MA, 02114, USA.; 4 Department of Radiation Oncology, Massachusetts General Hospital, Harvard Medical School, Boston, MA, 02114, USA.; 5 Department of Neurology and Cancer Center, Massachusetts General Hospital, Harvard Medical School, Boston, MA, 02114, USA.

## Abstract

**Rationale:** *NF2*-related schwannomatosis (*NF2*-SWN) is a progressive neurological disorder with a hallmark of bilateral vestibular schwannomas (VSs), leading to irreversible hearing loss and reduced quality of life. To date, the FDA has not approved any pharmacological therapies for treating VS or hearing loss. While radiotherapy (RT) is the standard treatment for growing VSs, it often exacerbates hearing loss. Immune checkpoint inhibitors (ICIs) have revolutionized cancer treatment; however, their efficacy in non-malignant tumors like VS remains largely unexamined.

**Methods:** We used immune-competent VS mouse models to assess the efficacy of combined anti-PD1 (αPD1) and RT treatment, tumor growth, and hearing preservation.

**Results:** We found three significant therapeutic benefits: i) RT induces immunogenic cell death and activates the STING pathway, enhancing αPD1 efficacy and generating long-term immune memory, ii) The combination strategy reduces the required RT dose necessary for effective tumor control, potentially minimizing RT injury to surrounding normal tissues, and iii) RT to peripheral nerve tumor induces a systemic abscopal effect, which enhances αPD-1 efficacy to effectively control intracranial schwannomas without direct irradiation, sparing the cochlea from radiation exposure and avoiding auditory radiation injury.

**Conclusion:** Our findings provide a compelling rationale for deploying ICIs in combination with radiotherapy as a novel treatment approach for patients with VS and *NF2*-SWN.

## Introduction

*NF2*-related schwannomatosis (*NF2*-SWN) is a dominantly inherited neoplasia syndrome caused by germline mutation in the *NF2* tumor suppressor gene. *NF2*-SWN occurs in approximately 1 in 61,000 individuals and exhibits an almost 100% penetrance 1, 2. Patients with *NF2*-SWN develop multiple non-malignant peripheral nerve sheath tumors, and the pathognomonic hallmark is bilateral vestibular schwannomas (VSs). The continuous growth of VSs results in sensorineural hearing loss (SNHL) and considerable morbidity, significantly impacting patients' quality of life 3. In advanced cases, large VSs can compress the brainstem, resulting in severe morbidity and even death 4. Standard treatments for growing VSs include surgery and radiotherapy (RT), however, both pose substantial risks, including irreversible hearing loss, persistent vestibular dysfunction, and cranial nerve impairment 5-7. There is a critical need for targeted pharmacologic therapies to arrest VS progression and mitigate SNHL.

In patients with sporadic VS, tumor control after irradiation is excellent, with long-term tumor control rates of around 88-91%, but significantly lower for patients with *NF2*-SWN 8, 9. In addition, radiotherapy can cause adverse effects, including: i) further hearing loss due to ototoxicity 8, 9, ii) pseudoprogression - a transient increase in tumor volume that occurs in 23-44% of VS patients and can worsen brainstem compression 10-12, and iii) increased risk of malignant transformation 13-15. There is a need for a combination regimen that can lower the therapeutic dose of RT required for tumor control, and, consequently, reduce radiation-induced ototoxicity and adverse effects.

Immune checkpoint inhibitors (ICIs) have revolutionized cancer therapy by enhancing the body's anti-tumor immune response. These agents, including monoclonal antibodies targeting programmed cell death protein-1 (PD-1), its ligand PD-L1, and cytotoxic T-lymphocyte-associated protein 4 (CTLA-4), function by blocking immune inhibitory signals that suppress T cell activation. By restoring T cell effector functions, ICIs promote tumor cell elimination and have been associated with significant improvements in clinical outcomes across multiple malignancies 16, 17. As a result, ICIs have received FDA approval to treat a broad spectrum of solid tumors, either as monotherapy or in combination with chemotherapy and other therapeutic modalities 18.

VS harbor substantial infiltration of CD4^+^ and CD8^+^ T cells 19; however, a large fraction of these tumor-infiltrating lymphocytes express PD-1 20, suggesting impaired immune function 21. There is high expression of PD-1 ligand, B7-H1, in VS 19, 22, highlighting the potential role of immune checkpoint pathways in the tumor microenvironment. Despite these observations, few studies have explored the treatment potential of ICIs in VS. In a subcutaneous mouse model, an anti-PD1 (αPD1) antibody modestly delayed schwannoma growth 23. In the clinical setting, a case report documented that αPD1 salvage therapy led to tumor growth arrest in a patient with recurrent VS 24. This observation demonstrates the therapeutic potential of ICIs for VS and warrants further investigation into their efficacy on tumor growth and hearing preservation.

In this study, we aim to address three questions: 1) Can the combination of αPD1 and RT treatment enhance anti-tumor efficacy compared to each monotherapy? 2) Can the addition of αPD1 treatment reduce the required RT dose for tumor control, thereby minimizing potential RT-related ototoxicity and other adverse effects? and 3) can local irradiation of a peripheral nerve schwannoma elicit an abscopal effect that synergizes with systemic αPD1 treatment and shrinks intracranial vestibular schwannoma without direct irradiation, thus sparing the cochlea from RT-related ototoxicity and better preserving hearing function?

## Materials and Methods

**Cell lines.** Mouse *Nf2*^-/-^ Schwannoma cells were maintained in 10% fetal bovine serum (FBS)-containing Schwann cell medium with Schwann cell growth supplement (SCGS, ScienCell) 25. Mouse SC4 Schwannoma cells (gift from Dr. Vijaya Ramesh, Massachusetts General Hospital, MGH) were maintained in 10% FBS-containing Dulbecco's Modified Eagle Medium (DMEM, Corning) 26, 27. Both tumor cell lines were infected with lentivirus encoding secreted Gaussia luciferase (Gluc) reporter gene to monitor tumor growth in the brain.

**Animal models.** All animal procedures were performed following the Public Health Service Policy on Humane Care of Laboratory Animals and approved by the Institutional Animal Care and Use Committee of the MGH. *Nf2^-/-^* and SC4 tumors were implanted in immune-competent C57/FVB mice. In all animal experiments, we used 8-12 weeks-old mice in an equal ratio of male and female mice (1:1) and used age- and sex-matched mice.

*Cerebellopontine angle (CPA) model:* To recapitulate the intracranial microenvironment of VSs, tumor cells were implanted into the CPA region of the right hemisphere 28, 29. A total of 1 μl of tumor cell suspension (2,500 cells) was implanted per mouse. To evaluate tumor growth in the brain, blood levels of secreted Gluc were measured as described before 29-32.

*Sciatic nerve schwannoma model:* To reproduce the microenvironment of peripheral schwannomas, we implanted tumor cells into the mouse sciatic nerve 26. A total of 3 μl of tumor cell suspension (5x10^4^ cells) was injected slowly (over 45-60 seconds) under the sciatic nerve sheath using a Hamilton syringe to prevent leakage. The sciatic nerve tumor size was measured using a caliper every 3 days until tumors reached 1 cm in diameter.

**Treatment protocols.** In the sciatic nerve model, treatment starts when the tumor reaches 3 mm in diameter. In the CPA model, treatment starts when the blood Gluc reaches 1x10^4^ RLU.

*Anti-PD1 treatment*. The anti-PD1 antibody or isotype control IgG (200 μg/mouse, Bioxell) was administered *i.p.* every 3 days for a total of 4 dosages.

*Radiation therapy.* Treatment was delivered in a ^137^Cs gamma irradiator for small animals, which produces 1.176 MeV gamma rays and allows longitudinal radiation studies. Mice were irradiated locally in a custom-designed irradiation chamber, which shields the entire animal except for the brain (in the CPA model) or the leg (in the sciatic nerve model) 26. We used 5 Gy or 10 Gy irradiation in our study based on a previously established protocol for mouse schwannoma models 26.

**Audiometric testing in animals.** Auditory brainstem responses (ABRs) were measured as described previously 29. Briefly, animals were anesthetized via i.p. injection of ketamine (0.1 mg/g) and xylazine (0.02 mg/g). The tympanic membrane and the middle ear were microscopically examined for signs of otitis media. All animals had well-aerated middle ears. ABRs were recorded between subdermal needle electrodes: positive in the inferior aspect of the ipsilateral pinna, negative at the vertex, and ground at the proximal tail. The responses were amplified (10,000X), filtered (0.3-3.0 kHz), and averaged (512 repetitions) for each of the same frequencies and sound levels. Custom LabVIEW software for data acquisition was run on a PXI chassis (National Instruments Corp). For each frequency, the auditory threshold was defined as the lowest stimulus at which repeatable peaks could be observed on visual inspection. In the absence of an auditory threshold, a value of 85 dB was assigned (5 dB above the maximal tested level).

**Analysis of tumor-infiltrating immune cells.** To characterize the tumor-infiltrating immune cells, tumor lysates were washed with PBS and stained for flow cytometry with anti-CD45 (clone 30-F11), anti-CD4 (clone RM4-5), anti-CD8 (clone 53-6.7), anti-Foxp3 (clone D6O8R), anti-NK1.1 (clone PK136), anti-Gr1 (clone RB6-8C5), anti-CD11b (clone m1/70), anti-F4/80 (clone BM8), anti-iNOS (clone CXNFT), anti-Arginase I (clone D4E3M), anti-CD86 (clone GL-1), anti-interferon (IFN-γ, clone XMG1.2), anti-TNFα (clone MP6-XT22), and anti-Granzyme B (clone GB11), see Supplementary Table 1 for antibody details.

**Gene expression analysis.** *RNASeq.* Fresh mouse tumor samples were homogenized using the polytron PT1300 tissue homogenizer, followed by additional homogenization using a Qiashredder spin column. RNA from tumor tissues was extracted using the RNeasy Mini Kit (QIAGEN, Cambridge, MA). 1 μg of total RNA was sent to the Molecular Biology Core Facilities, Dana-Farber Cancer Institute. RNASeq analysis was performed following the routine procedure 27. The DESeq2 package in R was used to determine the differentially expressed genes (DEGs) 33. To control for the False discovery rate (FDR) at 0.05, we used the Benjamini & Hochberg correction. The ComplexHeatmap package was used to plot the heatmap 34. The differentially expressed gene set was analyzed by Gene Set Enrichment Analysis software (GSEA, https://software.broadinstitute.org/software/cprg/?q=node/14).

*Quantitative RT-PCR.* qPCR was performed by SYBR Green methods as previously described 35, 36.

*Western Blot.* Western blot membranes were blotted with antibodies against total (1:500) and phospho-STING (1:1000); total (1:500) and phospho-TBK1 (Ser172, 1:1,000); total (1:500) and phospho-IRF3 (Ser396, 1:1,000); HMGB1 (1:500), cGAS (1:500) and beta-actin (1:5,000). The corresponding secondary antibodies were used. Antibodies were obtained from Cell Signaling (Danvers, MA) 37, 38.

*ELISA.* Plasma or tumor lysates (2 μg/μl concentration, triplicate for each sample) were analyzed using mouse multiplex enzyme-linked immunosorbent assay plates following the manufacturer's instructions (Meso-Scale Discovery, Gaithersburg, MD) 35, 39.

***In vitro* viability and apoptosis assay.** Cell viability was determined *in vitro* by MTT assay 40. In vitro apoptosis was determined by Annexin V-FITC and propidium iodide (PI) staining followed by flow cytometry, following the manufacturer's instructions (Biolegend).

**ATP release assay.** ATP release was evaluated using the Luminescent ATP Detection Kit (Abcam) following the manufacturer's instructions. Briefly, 50 µL of detergent was added to lyse the cells and stabilize the ATP. The plate was sealed and shaken for 5 minutes at 600-700 rpm. Then, 50 µL of substrate solution was added to each well, and luminescence was measured using a microplate reader. ATP levels were calculated by comparing luminescence values to a standard curve prepared using known ATP concentrations.

**Histological staining.** Tumor cell proliferation (PCNA^+^, 1:1,000, Abcam) and apoptosis (TUNEL^+^, ApopTag®, EMD Millipore) were evaluated by immunohistochemical staining 39. Archived paraffin-embedded patient VS samples were stained with antibodies against phosphorylated TBK (1:10, Cell Signaling Technology). Normal peripheral nerves (n=4) obtained postmortem were used as controls. Histological analysis with digital image quantification was conducted using ImageJ. Positive staining in 20 random fields/slides was quantified via automated functions based on fluorescent intensity, with a threshold to exclude background staining.

**Statistical analyses.** Differences in sciatic nerve tumor growth were analyzed using repeated measures two-way ANOVA. Survival curves were generated using the Kaplan-Meier method. Kaplan-Meier survival curves were analyzed by the Log-rank (Mantel-Cox) test. ABR thresholds were analyzed with a linear mixed-effects model. Flow cytometry, ELISA, qPCR, and cytotoxicity studies were analyzed using Student's t-test and Mann-Whitney U test, as appropriate. All statistical analyses were carried out using GraphPad Prism Software version 9.

## Results and Discussion

**Figure 1:**
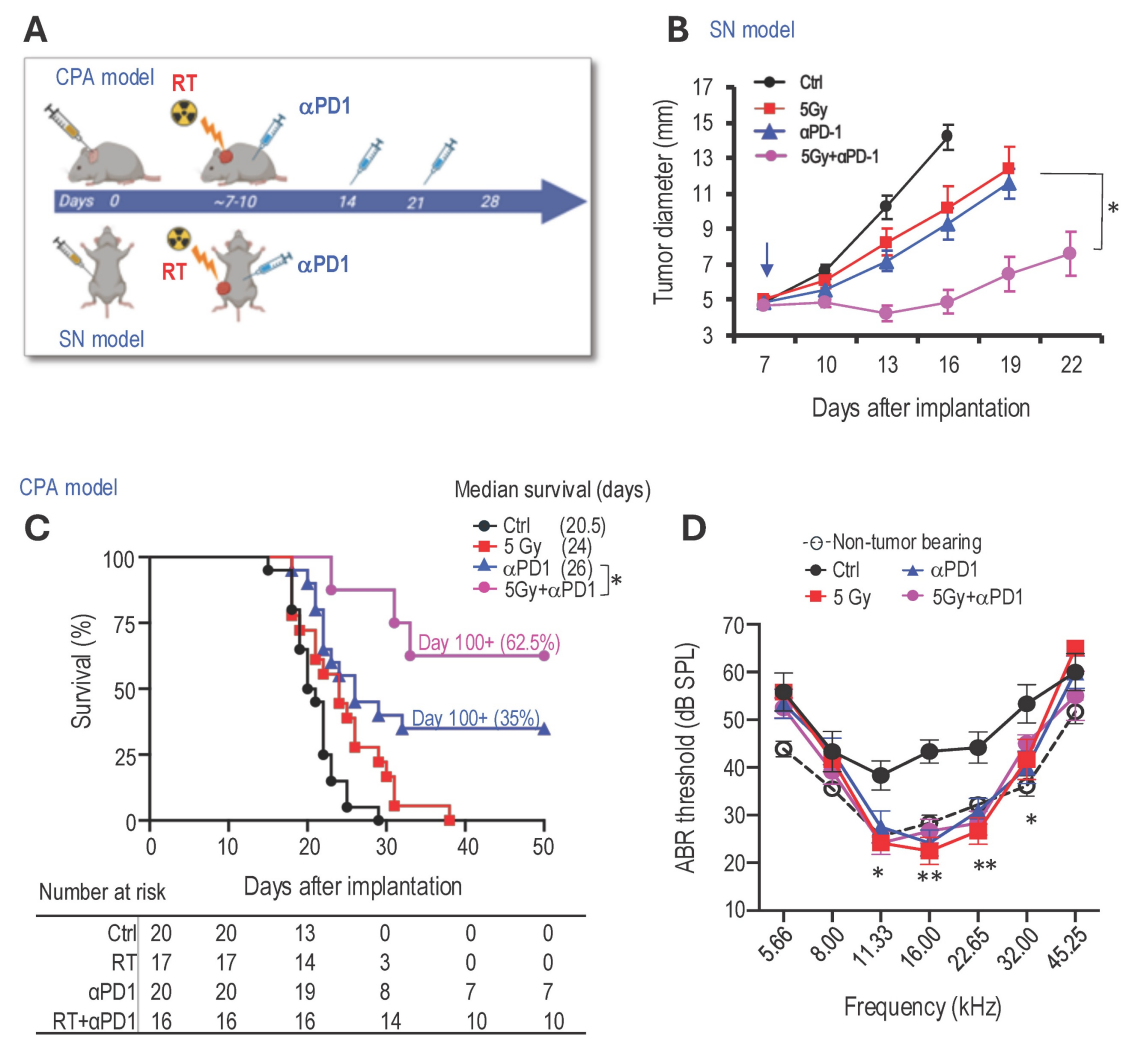
** Combined radiotherapy and αPD1 show improved tumor efficacy in both schwannoma models. (A)** Schematic and timeline of combined radiation therapy and αPD1 treatment in the CPA and SN schwannoma models. **(B)** Tumor diameter measured by caliper in the SN schwannoma model. Arrow, treatment start time. N=8/group, Data are presented as mean±s.e.m., and representative of at least three independent experiments, N=24 mice/group. **(C)** Kaplan-Meir survival curve of mice bearing *Nf2^-/-^* tumor in the CPA model. Ctrl (n=20), 5 Gy (n=17), αPD1 (n=20), Comb (n=16). **(D)** ABR threshold of mice bearing *Nf2^-/-^* tumor in the CPA model 21 days post-treatment. N=6/group, Data are presented as mean±s.e.m., and representative of at least three independent experiments, N=18 mice/group. Differences in sciatic nerve tumor growth was analyzed using repeated-measures two-way ANOVA. Kaplan-Meier survival curves were analyzed by Log-rank (Mantel-Cox) test. ABR thresholds were analyzed with a linear mixed-effects model. *P<0.01, **P<0.001.

**Combined radiotherapy and αPD1 show improved tumor control efficacy in both schwannoma models.** To address our first question of whether combined RT+αPD1 treatment can achieve enhanced efficacy, we treated mice bearing *Nf2^-/-^* tumors in both sciatic nerve and CPA models with: i) control IgG, ii) αPD1, iii) 5Gy RT, or iv) αPD1+5Gy RT (Figure 1A). In the sciatic nerve model, both RT and αPD1 monotherapy modestly delayed tumor growth, and combined RT+αPD1 treatment was significantly more effective in tumor growth inhibition compared to each monotherapy (Figure 1B). In the CPA model, combined RT+αPD1 treatment significantly prolonged survival - with 62.5% of mice surviving ≥100 days, compared to 32% of long-term survivor mice in the αPD1 monotherapy group (Figure 1C). This improved efficacy of the combination treatment was similarly observed in a second schwannoma model, SC4 (Supplemental Figure 1A).

The impact of immunotherapy on hearing has not been systematically assessed. To assess potential ototoxicity, we examined the effects of 5 Gy RT and combined 5Gy + αPD1 treatment in non-tumor-bearing mice by measuring auditory brainstem evoked response (ABR). We observed that neither 5 Gy RT nor the combined 5Gy + αPD1 treatment altered the ABR threshold in mice at both 1 day and 42 days post-treatment (Supplemental Figure 1B-D). In mice with *Nf2^-/-^* tumors in the CPA region, treatment with αPD1, 5 Gy RT, or their combination restored ABR threshold to levels comparable to those in non-tumor-bearing mice. However, no treatment is more effective than the others (Figure 1D).

**Figure 2:**
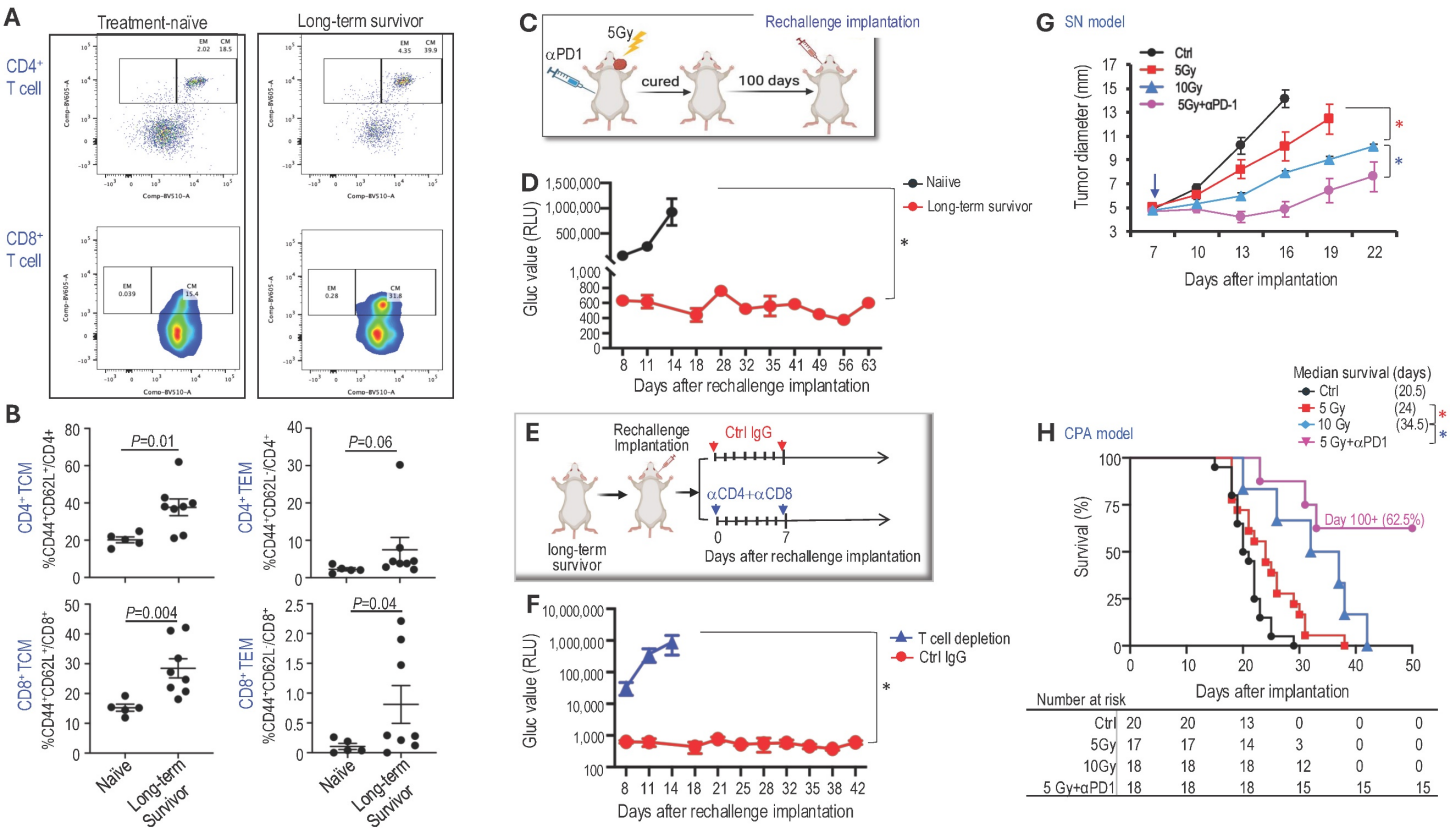
** Combined radiotherapy and αPD1 treatment generate immunologic memory and lowered the radiotherapy dose required for tumor control. (A)** Flow cytometry gating strategies to analyze CD4^+^ and CD8+ central memory T cells (TCM: CD44^+^CD62L^+^) and effector memory T cells (TEM: CD44^+^CD62L^-^) immune memories cells in treatment naïve and long-term survivors. **(B)** Flow cytometry quantification of CD4^+^ and CD8+ central memory and effector memory T cells in treatment naïve (n=5) and long-term survivors (n=8). **(C)** Schematic and timeline of rechallenging experiment in long-term survivors. **(D)** Blood Gluc level in naïve (n=8) and long-term survivors (n=8). Data are presented as mean±s.e.m., and representative of at least three independent experiments, N=24 mice/group. **(E)** Schematic and timeline of rechallenging experiment in long-term survivors with and without T cell depletion using anti-CD4 and anti-CD8 antibodies. **(F)** Blood Gluc level in long-term survivors treated with control IgG (n=12) or anti-CD4 and CD8 (n=12). Data are presented as mean±s.e.m. **(G)** Tumor diameter measured by caliper in the SN schwannoma model. Data are presented as mean±s.e.m., and representative of at least three independent experiments, N=24 mice/group. **(H)** Kaplan-Meir survival curve of mice bearing *Nf2^-/-^* tumor in the CPA model. Ctrl (n=20), 5 Gy (n=17), 10 Gy (n=18), 5Gy+αPD1 (n=18). Differences in sciatic nerve tumor growth was analyzed using repeated-measures two-way ANOVA. Kaplan-Meier survival curves were analyzied by Log-rank (Mantel-Cox) test. *P<0.01.

**Combined radiotherapy and αPD1 treatment generates immunologic memory.** To test whether long-term survivors develop durable immune memory, we first examined the memory T cell populations using flow cytometry (Figure 2A). Compared to treatment-naïve mice, long-term survivors showed significantly increased CD4^+^ and CD8^+^ central memory T cells (TCM: CD44^+^CD62L^+^), moderately increased CD4^+^ effector memory T cells (TEM: CD44^+^CD62L^-^), and significantly increased CD8^+^ TEM (Figure 2B).

Next, we re-challenged 'cured' mice (the initial tumor completely disappeared as confirmed by negative Gluc measurement, and mice survived over 100 days) with injections of *Nf2^-/-^*-Gluc cells into the CPA region contralateral to the previous injection (Figure 2C). Naïve mice were included as controls for tumor take. In naïve mice, 100% developed tumors, and their blood Gluc reporter gene levels exceeded 1x10^6^ RLU by day 14 after implantation. On the contrary, by day 63 after the rechallenge implantation, none of the 'cured' mice developed tumors (Figure 2D). These data suggest that the 'cured' mice have long-term tumor-specific immunological memory against the *Nf2^-/-^* cells.

Lastly, to evaluate whether T cells are critical for the anti-tumor immune memory in long-term survivors, a separate cohort of mice that had cleared tumors after combined therapy was rechallenged by implanting new tumors in the contralateral CPA region. Following rechallenge, mice were randomized to receive either control IgG or depleting antibodies against CD4 and CD8 T cells (Figure 2E). Long-term survivors treated with control IgG showed robust immune protection and failed to develop detectable tumors for more than 6 weeks after rechallenge. In contrast, T cell depletion abolished this protection, and mice rapidly developed progressive tumors (Figure 2F), demonstrating that T cells are required for maintaining long-term antitumor immunity in this model.

**Combined αPD1 treatment reduced the radiotherapy dose required for tumor control.** Key determinants of hearing loss after RT are the radiation dose and the volume of the cochlea irradiated, with lower radiation doses associated with better hearing preservation 41. To address our second question, whether combined αPD1 treatment helps lower the required RT dose for tumor control, thereby minimizing potential RT-related ototoxicity and adverse events, we treated groups of mice with i) control IgG, ii) 5 Gy RT, iii) 10 Gy RT, or iv) 5 Gy RT+PD1. In both sciatic nerve (Figure 2G) and CPA (Figure 2H) models, we found that high-dose RT (10 Gy) was more effective than low-dose RT (5 Gy) in delaying tumor growth and prolonging survival. However, when combined with αPD1 treatment, 5 Gy RT was more effective than 10 Gy RT alone. These data suggest that combining αPD1 treatment with RT could help lower the required RT dose for tumor control.

**Figure 3:**
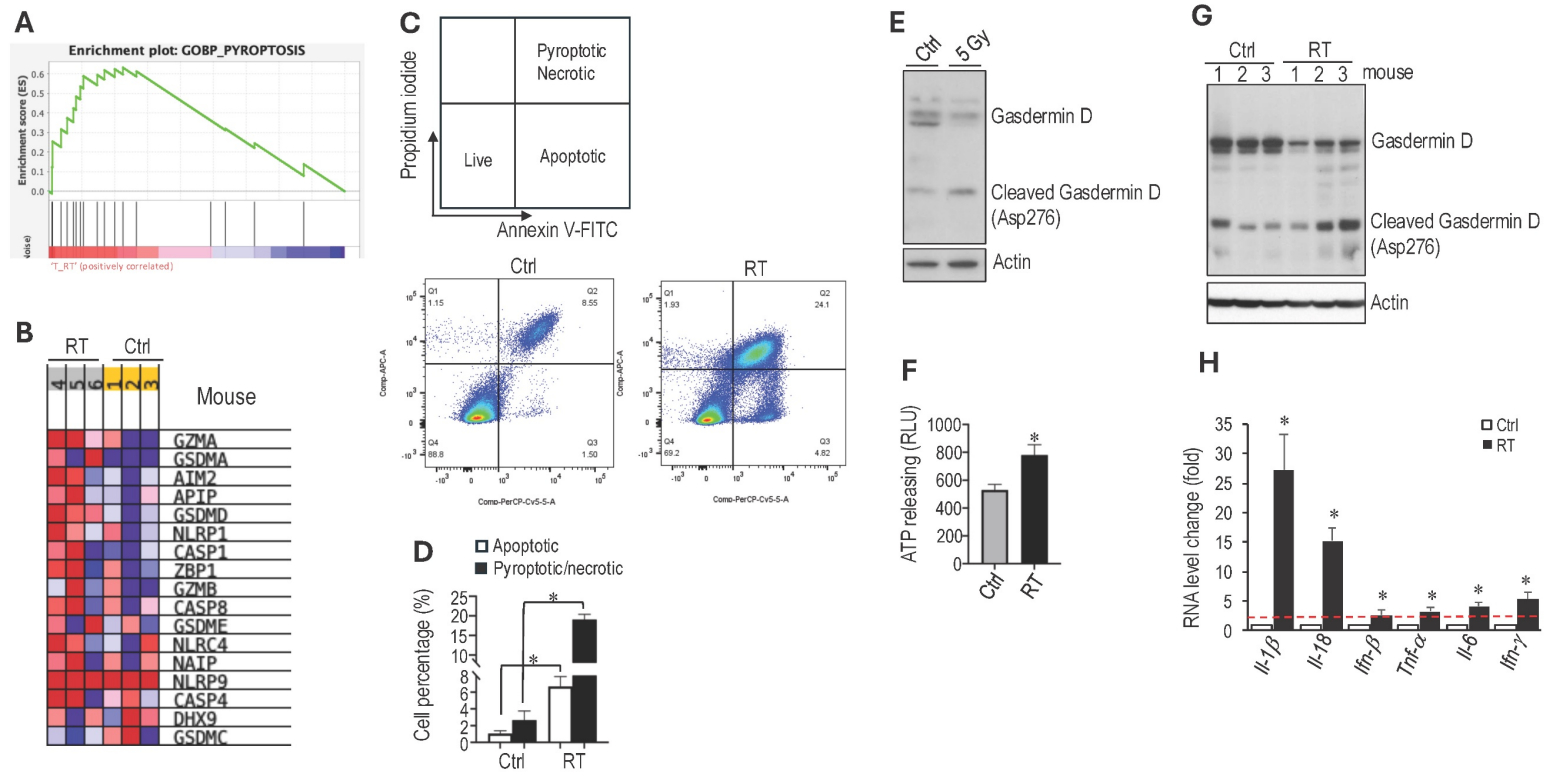
** RT induces immunogenic cell death in schwannoma models.** RNAseq analysis of bulk tumors from control and irradiated *Nf2^-/-^* tumors from the CPA mouse model (N=3 tumors/group). **(A)** GSEA enrichment plots of GO_Pyroptosis pathway related genes. **(B)** Heatmap of pyroptosis pathway genes. Flow cytometry of Annexin V/Propidium iodide-stained control and 5 Gy in vitro irradiated* Nf2^-/-^* cells. **(C)** Flow cytometry gating of Annexin V/Propidium iodide. **(D)** Flow cytometry analysis of apoptotic or pyroptotic and necrotic *Nf2^-/-^* cells. Confirmation of RT-induced Pyroptosis-related gene expression changes. **(E)** *Nf2^-/-^* tumor cells were treated with or without 5Gy irradiation. Six hours later, cells were lyse and protein was extracted for Western blot analysis of GasderminD. **(F)** Fluorescent intensity of extracellular ATP release from control and in vitro 5 Gy irradiated *Nf2^-/-^* cells (n=6 plates/group). **(G)** Western blot analysis of GasderminD in control and 5 Gy irradiated *Nf2^-/-^* tumor tissues. **(H)** qRT-PCR analysis of murine *Il-1β, Il-18, Ifn-β, Tnf-α, Il-6 and Ifn-γ* mRNAs in control and 5 Gy irradiated *Nf2^-/-^* tumors (N=3 tumors/group). Flow cytometry and gene expression data are presented as mean±s.d., and analyzed using Student's t-test and the Mann-Whitney test. *P<0.01.

**RT induces immunogenic cell death in schwannoma models.** Using RNASeq analysis to screen for changes in gene expression, we found that irradiation of *an Nf2^-/-^* tumor led to a significant upregulation of genes involved in pyroptosis (Figure 3A). Irradiated tumors showed increased gene expression of i) pyroptosis executors, including *Gasdermin D (Gsdmd)* and Gasdermin E (*Gsdme*), ii) caspases that cleave Gasdermin D, such as Caspase 1 (*Casp1*) and Caspase 4 (*Casp4*), and iii) inflammasomes, upstream activators of pyroptosis, including *Nlrp1* and *Nlrc4* (Figure 3B). Because pyroptosis is a form of immunogenic cell death (ICD) that can trigger robust systemic immune activation 42,43, these findings prompted us to investigate whether radiation therapy induces pyroptosis in our schwannoma models.

To determine whether radiation therapy induces pyroptosis, we first irradiated *Nf2^-/-^* cells *in vitro* and assessed cell death by flow cytometry (Figure 3C). Irradiation significantly increased tumor cell pyroptosis and necrosis (Figure 3D). To distinguish pyroptosis from necrosis, we evaluated Gasdermin D, the key executioner of pyroptosis 44, 45. In irradiated *Nf2^-/-^* cells, western blot analysis demonstrated a shift from full-length to cleaved Gasdermin D, marked by reduced full-length protein and the appearance of the cleaved fragment (Figure 3E). Because adenosine triphosphate (ATP) is both a trigger for and a product of pyroptosis 46, we next measured extracellular ATP levels and found that irradiated *Nf2^-/-^* cells released significantly more ATP compared to control cells (Figure 3F).

We further confirmed *in vivo* that RT increased the level of cleaved Gasdermin D by western blot (Figure 3G) and upregulated several inflammatory cytokines by quantitative RT-PCR, including IL-1β and IL-18, which are canonical cytokines released during pyroptosis, as well as IFN-α, TNF-α, IL-6, and IFN-γ, which reflects the inflammatory response associated with pyroptotic cell death (Figure 3H). Together, these data demonstrate that RT activates a pyroptosis-associated inflammatory cell death program in the schwannoma models.

**Figure 4:**
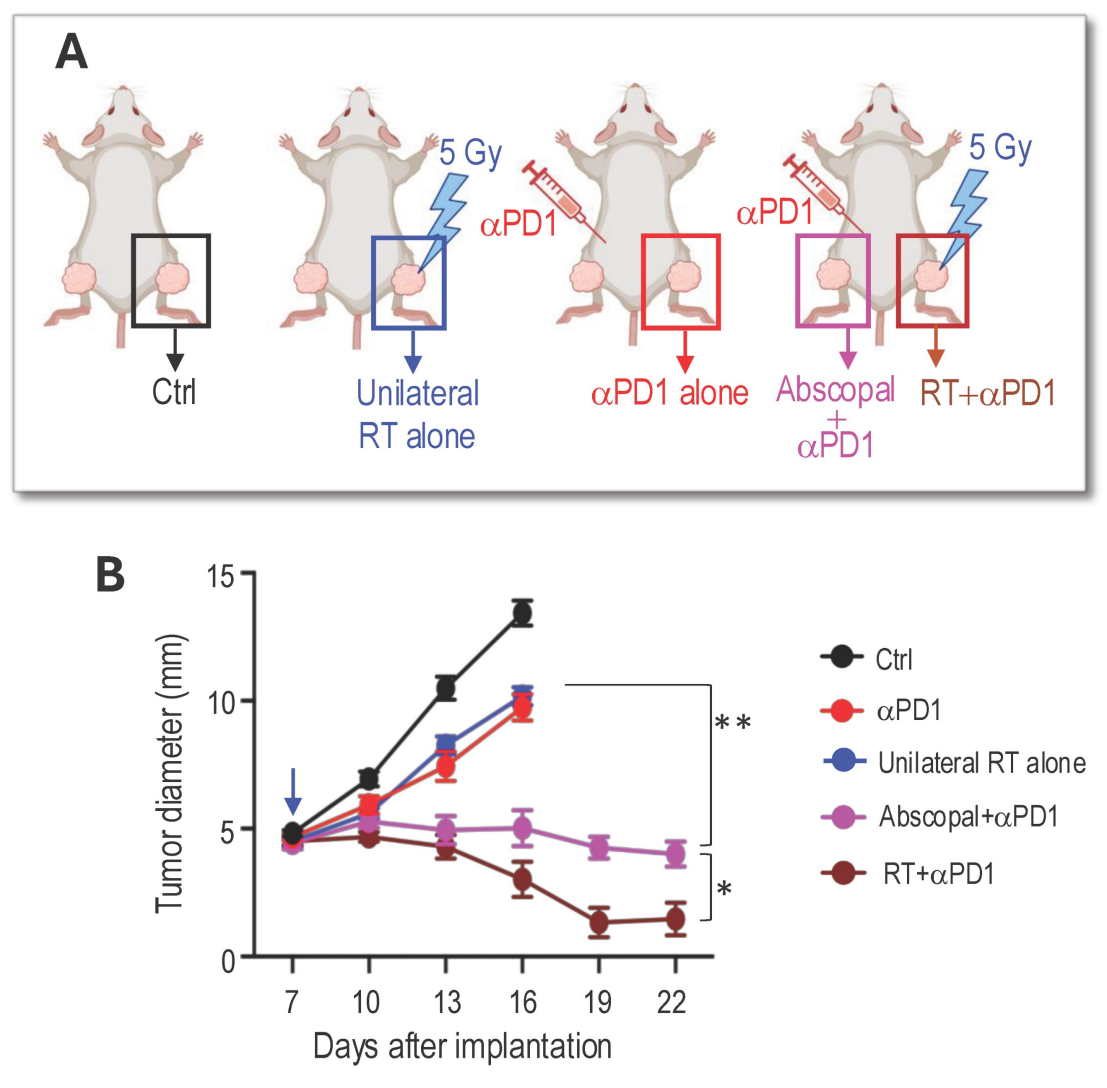
** RT of peripheral nerve schwannoma elicits an abscopal effect and additively with aPD1 efficacy. (A)** Schematic of bilateral *Nf2^-/-^* tumor implantation, systemic αPD1 treatment, and local radiation therapy to the right sciatic nerve schwannoma. **(B)** Tumor diameter measured by caliper. All animal studies are presented as mean±s.e.m., N=24 mice/group. Differences in sciatic nerve tumor growth was analyzed using repeated-measures two-way ANOVA. *P<0.01. ** P<0.001.

**RT of peripheral nerve schwannoma elicits an abscopal effect and enhances αPD1 efficacy.** We next performed an experiment to address our third question: whether local irradiation of a peripheral nerve schwannoma induces an abscopal effect that synergizes with systemic αPD1 treatment, leading to the shrinkage of intracranial vestibular schwannoma without direct irradiation. This approach aims to spare the cochlea from RT-related ototoxicity and better preserve hearing function. We bilaterally implanted *Nf2^-/-^* cells into the sciatic nerves of mice. When tumors grew to ~5 mm in diameter, mice were randomized into groups receiving control IgG or treatment with i) αPD1 only, ii) 5 Gy RT applied to the tumor on the right leg (unilateral RT alone), or iii) αPD1+5 Gy RT applied only to the tumor on the right leg, while the rest of the body was shielded (Figure 4A). Results indicated that while the group with the tumor receiving both direct RT and αPD1 had the best response (RT+ αPD1, brown line), the contralateral non-irradiated tumor receiving systemic αPD1 treatment plus the RT abscopal effect (Abscopal+αPD1, pink line) showed significantly reduced growth compared to tumors receiving either αPD1 (red line) or unilateral RT alone (blue line, Figure 4B).

**Figure 5:**
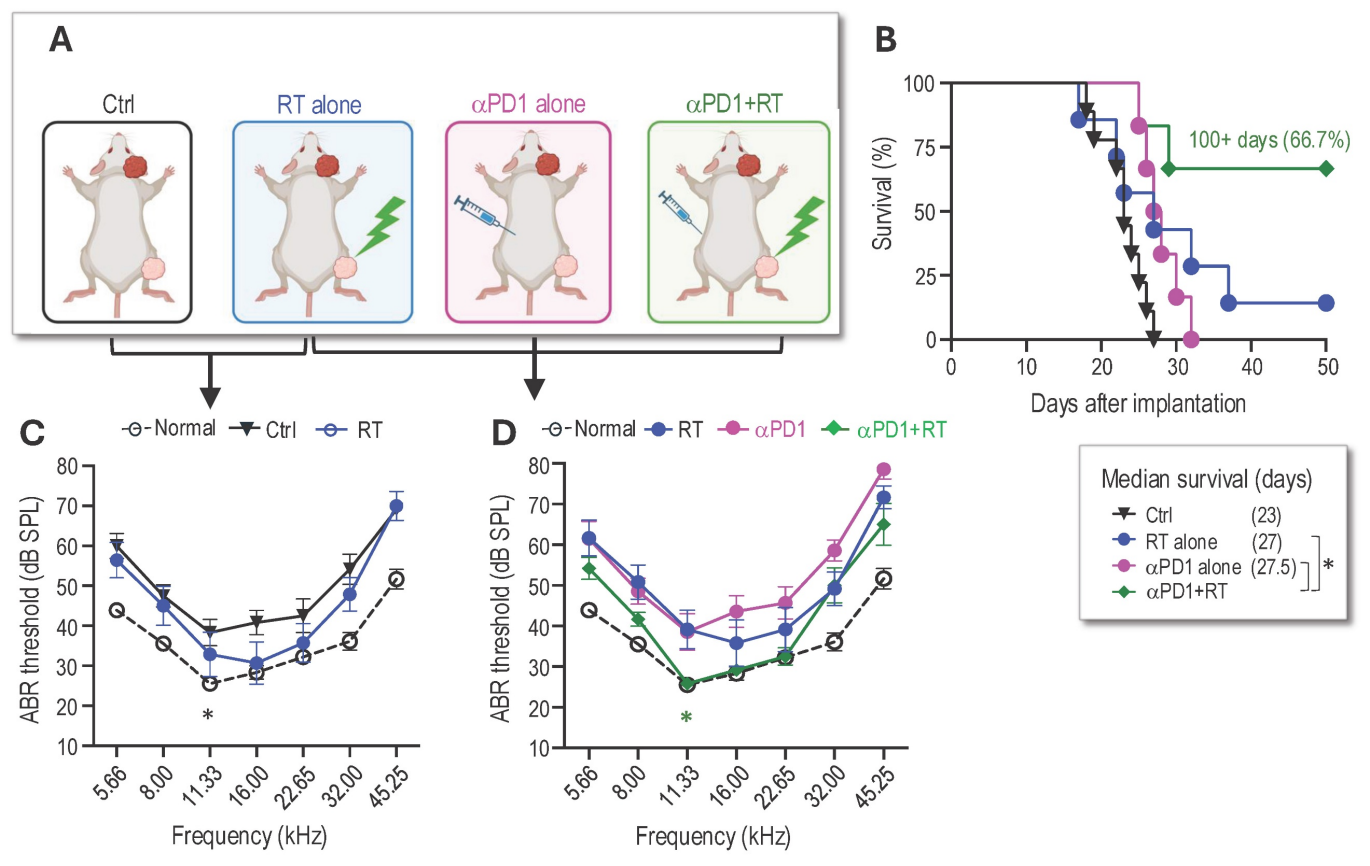
** RT of peripheral nerve schwannomas works additively with systemic αPD1 treatment to control intracranial tumors and preserve hearing. (A)** Schematic of local irradiation of the sciatic nerve *Nf2^-/-^* tumor and systemic αPD1 treatment in mice bearing *Nf2^-/-^* CPA tumors. **(B)** Kaplan-Meir survival curve of mice. **(C)** ABR threshold of the ear ipsilateral to tumor implantation in non-tumor bearing (black dotted line), control (black line), and 5 Gy RT alone (blue line) groups. **(D)** ABR threshold of the ear ipsilateral to tumor implantation in non-tumor bearing mice (black dotted line), and in mice receiving 5Gy RT alone to the SN tumor (blue line), *i.p.* injection of αPD1 alone (pink line), or 5 Gy to the SN tumor + *i.p.* injection of αPD1 (green line). *P<0.01 for green vs. blue and green vs. pink. All animal studies are presented as mean±s.e.m., N=18 mice/group, and representative of at least three independent experiments. Kaplan-Meier survival curves were analyzed by Log-rank (Mantel-Cox) test. ABR thresholds were analyzed with a linear mixed-effects model. *P<0.01.

**RT of peripheral nerve schwannomas works additively with αPD1 to control intracranial tumors and preserve hearing.** Based on the observed abscopal effects, we next investigated whether local irradiation of the sciatic nerve tumor could synergize with systemic αPD1 treatment to inhibit intracranial tumor growth and prevent hearing loss. In this study, we implanted tumors in both the sciatic nerve and the CPA area. When the sciatic nerve tumors reached ~5 mm in diameter, the mice were randomized into control or treatment groups of i) 5 Gy RT to the sciatic nerve tumor alone, ii) αPD1 alone, or iii) a combination of αPD1+5 Gy RT to the sciatic nerve tumor (Figure 5A). Mice were sacrificed when they exhibited ataxia, a symptom caused by the CPA tumors. Compared to the control group, local irradiation of the sciatic nerve tumor extended animal survival by 4 days (Figure 5B) and prevented tumor-induced hearing loss (Figure 5C). The RT abscopal effect, when combined with systemic αPD1 treatment, significantly prolonged mice survival, with 66.7% of animals surviving over 100 days (Figure 5B). More importantly, we found that irradiation of the sciatic nerve tumor synergized with systemic αPD1 treatment, more effectively preventing tumor-induced hearing loss than αPD1 monotherapy (Figure 5D).

**Figure 6:**
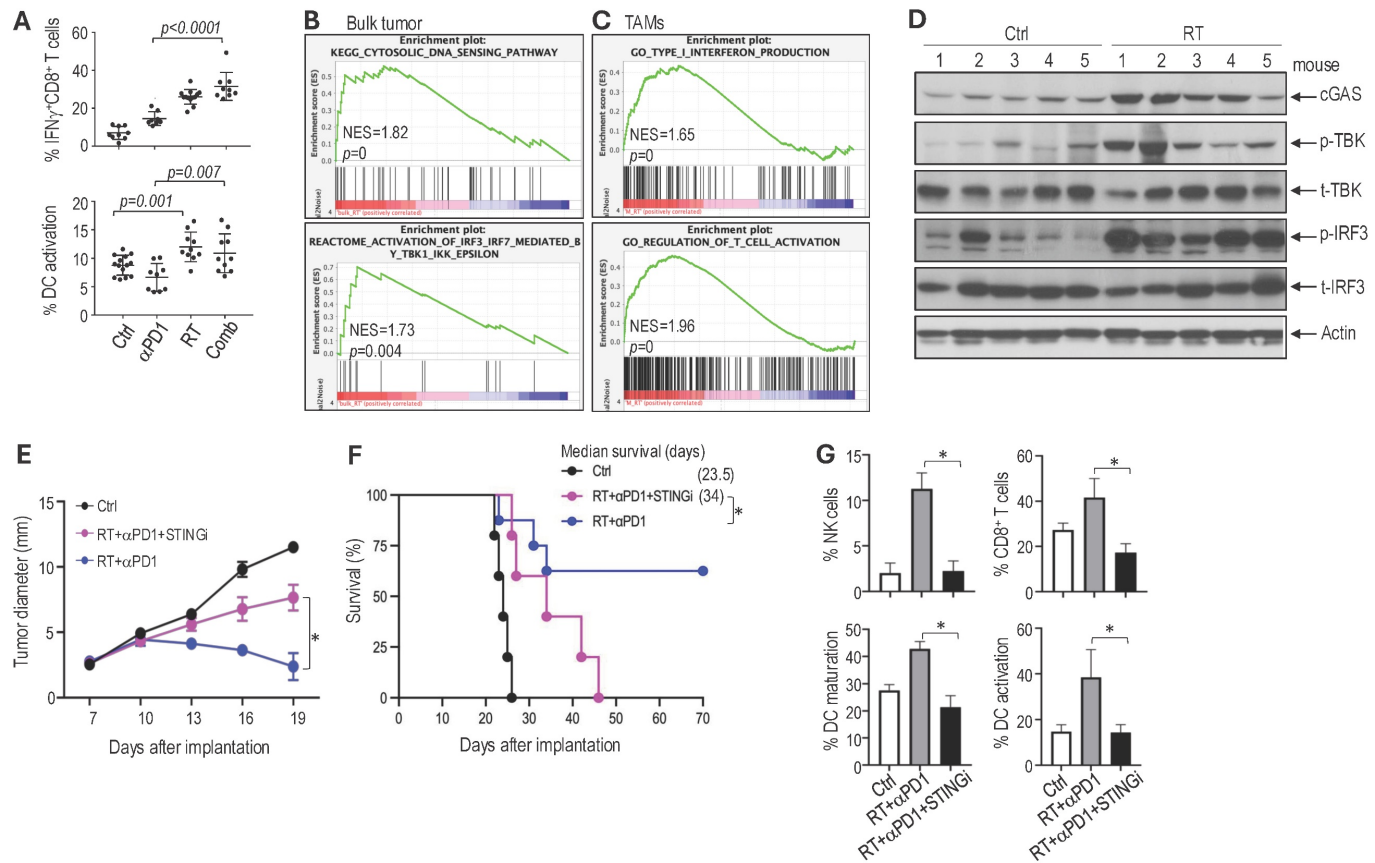
** STING signaling mediates the therapeutic benefits observed in the combined RT and aPD1 treatment in Schwannoma models. (A)** Flow cytometry analysis of IFNg expressing CD8^+^ T cells (% of IFNg^+^ CD8 T cell/CD8^+^CD3^+^CD45^+^) and activated dendritic cells (% of CD86^+^CD11c^+^CD11b^low/-^CD45^+^/CD11c^+^CD11b^low/-^CD45^+^) and in *Nf2^-/-^* tumors. **(B)** GSEA enrichment plots comparing irradiated vs. non-irradiated control bulk *Nf2^-/-^* tumors. **(C)** GSEA enrichment plots of TAMs isolated from irradiated vs. non-irradiated control *Nf2^-/-^* tumors. **(D)** Western blot analysis of STING pathway in tumor tissues (N=5 tumors/group). **(E)** Tumor diameter measured by caliper. **(F)** Kaplan-Meir survival curve of mice. **(G)** Flow cytometry analysis of NK cells, CD8 T cells, and dendritic cell maturation and activation in *Nf2^-/-^* tumors. All animal studies are presented as mean±s.e.m., N=24 mice/group, and representative of at least three independent experiments. Differences in sciatic nerve tumor growth was analyzed using repeated-measures two-way ANOVA. Kaplan-Meier survival curves were analyzed by Log-rank (Mantel-Cox) test. Gene expression and flow cytometry data are presented as mean ± s.d., and analyzed using Student's t-test and the Mann-Whitney test. *P<0.01.

**Combined radiotherapy and αPD1 treatment activates dendritic cells and CD8 T cells.** Compared to the αPD1 monotherapy, the combination with RT resulted in a significant increase in intratumoral CD8^+^ T cells expressing interferon (IFN)-γ, a key cytokine produced by activated CD8^+^ T cells that is essential for antitumor immunity. Dendritic cells (DCs) are critical for priming CD8^+^ T cell responses; therefore, we evaluated DC activation state following treatment. Quantification of activated DCs showed that αPD1 monotherapy did not increase DC activation compared to control. In contrast, RT alone increased the intratumoral DCs expressing CD86, a costimulatory molecule essential for activating T cells. The combination of RT with αPD1 resulted in similarly elevated levels of DC activation. These data indicate that RT enhances antitumoral antigen presentation and promotes further activation of CD8 T cells (Figure 6A). The RT-induced increase in tumor-infiltrating T cells was further confirmed by immunofluorescent analysis (Supplemental Figure 2A). Consistent with increased immune activation, CPA tumors from mice in the combination treatment group exhibited a higher number of apoptotic tumor cells (TUNEL^+^) and a reduced number of proliferating tumor cells (PCNA^+^) compared to those in the control or monotherapy groups (Supplemental Figure 2B-C).

**STING signaling mediates the therapeutic benefit observed with combined RT and αPD1 treatment in Schwannoma models.** We next investigated the molecular mechanisms underlying the therapeutic benefit of the combination treatment. Using RNASeq, we profiled gene expression changes in both *Nf2^-/-^* bulk tumors and in tumor-associated macrophages (TAMs). In the *Nf2^-/-^* bulk tumor, RT enriched gene signature associated with cytosolic DNA sensing and IRF3-TBK1 pathways (Figure 6B). In TAMs isolated from *Nf2^-/-^* tumors, RT induced enrichment of type I IFN and T cell activation pathways (Figure 6C). RT-induced DNA damage activates the cytosolic DNA-sensing pathway mediated by cGAS and stimulator of interferon genes (STING). STING recruits TBK1 and activates IRF3 to induce the expression of type I interferon (IFN), which activates the immune response 47, 48, and is critical for immune checkpoint inhibitor therapy and radiotherapy 49-52.

Using western blot analysis, we confirmed in the CPA tumors that RT induced the expression of cyclic GMP-AMP (cGAMP) synthase (cGAS), phosphorylation of TANK-binding kinase 1 (TBK1), and interferon regulatory factor 3 (IRF3)(Figure 6D). As the single-cell RNASeq data and bulk tumor signal transduction analysis converge on DNA sensing, IRF3-TBK, and IFN production, we focused on the STING pathway.

To investigate whether STING signaling contributes to the therapeutic benefit of the combination treatment, we treated mice bearing *Nf2^-/-^* tumors in the sciatic nerve with i) control, ii) RT+αPD1, and iii) RT+αPD1+selective STING inhibitor (H151). We found that the addition of the STING inhibitor abolished the therapeutic benefits of the combination treatment: i) H151 significantly reduced the tumor growth delay achieved by the combination therapy (Figure 6E), ii) shortened median survival to 34 days, compared to the 62.5% long-term survival rate observed in the combination treatment group (Figure 6F), and iii) abolished the combination treatment-induced recruitment of immune effector NK cells and CD8^+^ T cells as well as the activation of dendritic cells (Figure 6G). These data confirm that STING signaling contributes to the therapeutic benefit of the RT and αPD1 combination treatment.

## Discussion

RT is the standard of care for progressive or symptomatic vestibular schwannomas 53. Although fractionated radiation therapy has excellent tumor control, patients typically experience hearing deterioration within six months of radiation therapy, suggesting that hearing loss results from RT-induced ototoxicity rather than tumor progression 41. The goal of our study is to develop a therapeutic strategy that spares the cochlea from direct irradiation to minimize auditory radiation injury and better preserve hearing function in patients with *NF2*-SWN.

Multiple preclinical studies have shown that radiotherapy stimulates antitumor immunity through several complementary mechanisms: i) increasing tumor immunogenicity by increasing neoantigen expression and antigen presentation 54, ii) reprogramming the immunosuppressive tumor microenvironment by inducing immunostimulatory cytokines such as type I interferon 55, and iii) promoting the recruitment of antigen-presenting cells and immune effector cells to the tumor microenvironment 56. Consistent with these effects, numerous preclinical studies have demonstrated that combining radiotherapy with immune checkpoint inhibitors yields synergistic antitumor responses 57, 58. These findings provide a strong rationale for RT-ICI combinations, which may not only enhance therapeutic efficacy but also enable reduced radiation doses to achieve tumor control and potentially lower the risk of RT-related ototoxicity.

In our studies, we demonstrated that the combination of αPD1 treatment with RT provides three significant benefits: i) enhanced αPD1 efficacy and immune memory, ii) reduced RT dose and associated tissue injury, and iii) elicited abscopal effects on intracranial schwannomas, sparing the cochlea from radiation exposure and avoiding auditory radiation injury. The novelty of our work is twofold: i) first systematic evaluation of immunotherapy in a non-malignant tumor: To our knowledge, this is the first comprehensive assessment of immunotherapy in a non-malignant tumor setting, with dual emphasis on both tumor control and preservation of neurological function (hearing); and ii) clinically relevant innovation for vestibular schwannoma: We identified a potential strategy to avoid direct cochlear irradiation, which may help reduce radiation-induced auditory injury - a clinical issue uniquely relevant to vestibular schwannoma management and not addressable in prior malignant tumor studies.

Our first finding is that RT, by inducing immunogenic cell death and activating the STING pathway, enhances the efficacy of αPD1 treatment. Although immunotherapy has transformed cancer treatment, its efficacy as monotherapy is still limited, with ICIs providing benefits to only 20-30% of patients with non-small cell lung cancer (NSCLC), renal cell carcinoma, or melanoma 59. To date, indications for ICIs have often combined treatment strategies to enhance efficacy. In addition to its direct tumoricidal effects, focal RT can trigger systemic anti-tumor immunity by: i) promoting *in situ* vaccination through the release of tumor-associated neoantigens 60, ii) activating dendritic cells to enhance cancer cell recognition by cytotoxic T lymphocytes (CTLs) 52, and iii) inducing immunogenic cell death 61. Combined RT and immunotherapy have shown increased efficacy in mouse models of colorectal cancer 62, breast cancer 57, pancreatic tumor 63; however, this strategy has not been evaluated in non-malignant schwannoma models.

In our VS model, we observed that RT induced immunogenic cell death, a form of regulated cell death that is sufficient to activate an adaptive immune response 64. Specifically, irradiation led to the release of immunostimulatory damage-associated molecular patterns (DAMPs), including ATP and HMGB1, from irradiated, dying tumor cells. At the molecular level, we found that RT activated the cGAS/STING pathway, leading to increased production of type I interferon, TNFα, and IL-1β. These RT-induced immune mediators may support αPD1 therapy to enhance its efficacy. Future genetic studies, such as siRNA-mediated knockdown or CRISPR-based gene editing of ICD genes (HMGB1, Gasdermin D, IL-1β, and IL-18), and key STING pathway members (cGAS, TBK, and IRF3) - will be crucial in definitively establishing the functional roles of these pathways and determining which molecular pathway drives the therapeutic benefit. Despite encouraging preclinical data on the improved efficacy of RT and αPD1 combination therapy, clinical studies have yielded mixed results. Maintenance αPD1 treatment following standard-of-care chemoradiotherapy has been shown to enhance overall survival (OS) in patients with NSCLC 65, 66; neoadjuvant stereotactic body radiotherapy (SBRT) combined with durvalumab (an aPD-L1 antibody) increases durvalumab response in early-stage NSCLC 67, while nivolumab (an aPD1 antibody) improves disease-free survival in NSCLC patients who received neoadjuvant chemoradiotherapy 68. Conversely, clinical studies in glioblastoma and head & neck squamous cell carcinomas failed to demonstrate a therapeutic benefit over RT or immunotherapy alone 69-72. Our findings provide a rationale for future clinical trials of this combination strategy in patients with VS, suggesting that optimally combining RT with immunotherapy may be a key to unlocking the potential of both therapies.

Secondly, we found that combined αPD-1 blockade can reduce the RT dose required for effective tumor control. Radiation therapy is known to cause permanent sensorineural hearing loss in a dose-dependent, progressive manner 73, and RT can also cause conductive hearing loss due to ear canal stenosis 74. Developing combination treatment regimens that reduce the RT dose while maintaining clinical efficacy is essential to minimize ototoxicity in VS therapies. In our VS model, we found that adding an αPD1 treatment reduced the RT dose required to control tumor growth, suggesting that this combination strategy could help mitigate RT-induced ototoxicity. These findings support further clinical investigation of combined RT and αPD1 treatment in patients with VS to enhance therapeutic outcomes and preserve hearing function. One limitation of our preclinical studies is that mouse tumors grow more rapidly than patient schwannomas, which limits our ability to investigate long-term radiation-induced ototoxicity. Several generations of *Nf2* tumor suppressor gene knockout mice have been developed, but earlier models failed to produce schwannomas characteristic of *NF2*-SWN 75. More recently, an improved conditional knockout model using tissue-restricted *Nf2* gene excision (*Postn-Cre;Nf2^flox/flox^*) was generated. These mice develop microscopic Schwann cell hyperplasia at 3-4 months, macroscopic Schwannomas along spinal, peripheral, and cranial nerves by 6-8 months, and exhibit hearing and balance deficits 76. This slower and more physiological growth course would be ideal for studying long-term treatment effects. Future efforts to use these mice will be necessary for better recapitulating the slow-growing, non-malignant nature of schwannomas.

Lastly, as *NF2*-SWN patients often develop schwannomas throughout the body, we simultaneously implanted schwannoma cells in the CPA and in the sciatic nerve in mice. We observed that irradiation of a peripheral nerve tumor induced an abscopal effect that synergized with systemic αPD1 treatment, effectively controlling intracranial tumor growth and, more importantly, preventing tumor-induced hearing loss without the need for direct irradiation of the CPA tumor. This strategy holds substantial clinical potential. Hypo-fractionated RT can elicit an abscopal effect, limiting the progression of distant tumors outside the locally irradiated area 77, and synergizing with ICI in both preclinical breast cancer, colorectal cancer, melanoma, and lung cancer mouse models 57, 78-80, and lung cancer and melanoma patients 81, 82. The cochlea is particularly sensitive to RT compared to either the brain or the auditory nerve 83, 84. Direct irradiation of the cochlea can damage critical auditory structures, including the organ of Corti, basilar membrane, spiral ligament, and stria vascularis, as well as causing outer hair cell loss in the basal turn. This damage is most manifested in the high-frequency hearing loss observed in patients 85. Our findings suggest that targeting peripheral nerve tumors while avoiding direct cochlear irradiation could reduce treatment-related side effects and better preserve hearing. This combination approach represents a promising shift toward more personalized, less toxic VS treatments. Additional human model studies will be essential for advancing our preclinical findings toward clinical translation. However, generating such data is challenging as VS is a rare disease, and surgical samples - particularly after radiotherapy - are extremely limited. Future studies with larger, multi-institutional patient cohorts will be critical for validating the immune-modulatory effects of radiotherapy. Furthermore, future studies incorporating patient-derived schwannoma cell lines using humanized mouse models will provide further support for advancing combined RT and αPD1 therapy toward clinical evaluation in VS.

## Conclusions

In summary, our studies demonstrate that combining αPD1 treatment with RT significantly improves the efficacy of both therapies. Combined RT enhances the effectiveness of αPD1 therapy by promoting immunogenic cell death and activating the STING pathway, while combined αPD1 therapy allows for a reduced RT dose, potentially minimizing RT-induced ototoxicity and other adverse effects. Most importantly, RT induces an abscopal effect that effectively controls intracranial tumors without directly irradiating the cochlea. This approach has the potential to protect the cochlea from radiation exposure, thereby preventing auditory damage and preserving hearing function. Our findings highlight the therapeutic potential of combining RT and αPD1 to optimize tumor control while minimizing treatment-related side effects, paving the way for more targeted and patient-centered cancer therapies.

## Supplementary Material

Supplementary figures and table.

## Abbreviations

ABR: Auditory brainstem response
αPD1: anti-PD1
Casp: Caspase 1
cGAS: Cyclic GMP-AMP (cGAMP) synthase
CPA: Cerebellopontine angle
CTL: Cytotoxic T lymphocytes
CTLA-4: Cytotoxic T-lymphocyte-associated protein 4
DAMP: Damage-associated molecular patterns
DEG: Differentially expressed gene
DMEM: Dulbecco's Modified Eagle Medium
FBS: Fetal bovine serum
FDR: False discovery rate
Gsdmd: Gasdermin D
GSEA: Gene Set Enrichment Analysis
Gluc: Gaussia luciferase
ICI: Immune checkpoint inhibitor
IRF3: Interferon regulatory factor 3
*NF2*-SWN: *NF2*-related schwannomatosis
NSCLC: Non-small cell lung cancer
OS: Overall survival
PD-1: Programmed cell death protein-1
PI: Propidium iodide
RT: Radiotherapy
SBRT: Stereotactic body radiotherapy
SCGS: Schwann cell growth supplement
SNHL: Sensorineural hearing loss
TBK1: TANK-binding kinase 1
TCM: Central memory T cells
TEM: Effector memory T cells
VS: Vestibular schwannoma
