# Supplementary material for: A cochlea-sparing strategy for non-invasive control of intracranial schwannomas via peripheral irradiation and anti-PD-1 therapy enhanced by STING activation
Source: Theranostics. 2026 Jan 21;16(8):4076–89. doi: 10.7150/thno.123726 (PMC12905750; doi:10.7150/thno.123726)
Supplement: Supplementary file 1 — Supplementary figures and table. [file thnov16p4076s1.pdf]

A Cochlea-Sparing Strategy for Non-Invasive Control of Intracranial Schwannomas via Peripheral Irradiation and Anti–PD-1 Therapy Enhanced by STING Activation

Authors

Zhenzhen Yin[1,a], Simeng Lu[1,a], Limeng Wu[1], Yao Sun[1,2], Day Caven Blake[1], Jie Chen[1], Lukas D. Landegger[3], William Ho[1], Bingyu Xiu[1], Adam P. Jones[1], Alona Muzikansky[4], Helen A. Shih[5], Konstantina M. Stankovic[3], Scott R. Plotkin[6], and Lei Xu[1,*]

Supplementary Figures

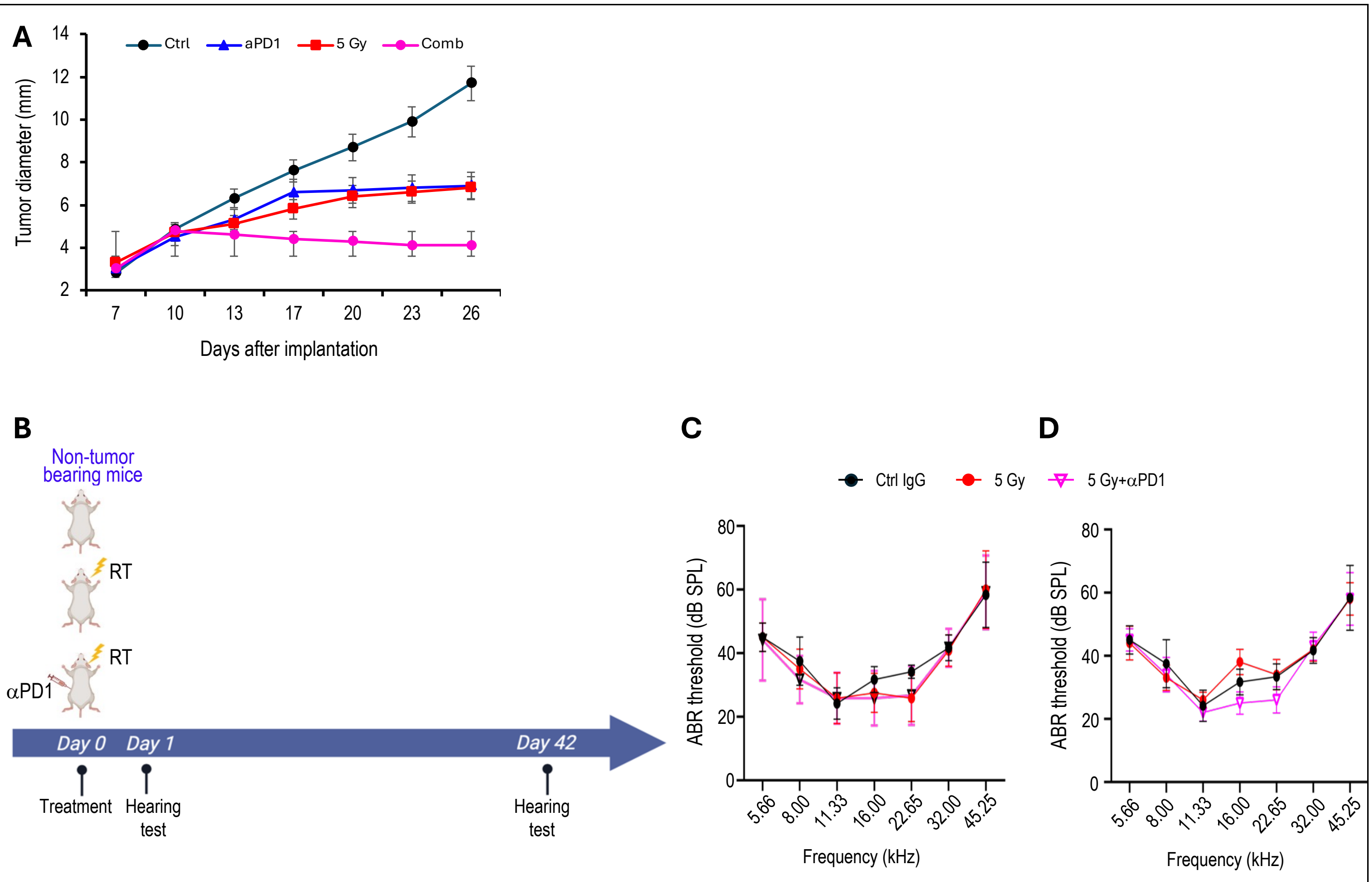

Figure S1. Combined radiation therapy enhances αPD1 efficacy in the mouse schwannoma models.

(A) Tumor diameter measured by caliper in the SC4 SN schwannoma model.

(B) Schematic and timeline of combined radiation therapy and αPD1 treatment in the CPA schwannoma models.

(C) Day 1 post-treatment ABR threshold of mice bearing *Nf2*$^{-/-}$ tumor in the CPA model.

(D) Day 42 post-treatment ABR threshold of mice bearing *Nf2*$^{-/-}$ tumor in the CPA model.

All animal studies are presented as mean±s.e.m., N=24 mice/group, and representative of at least three independent experiments. In vivo study significance was analyzed using a Student's t-test. *P<0.01, **P<0.001.

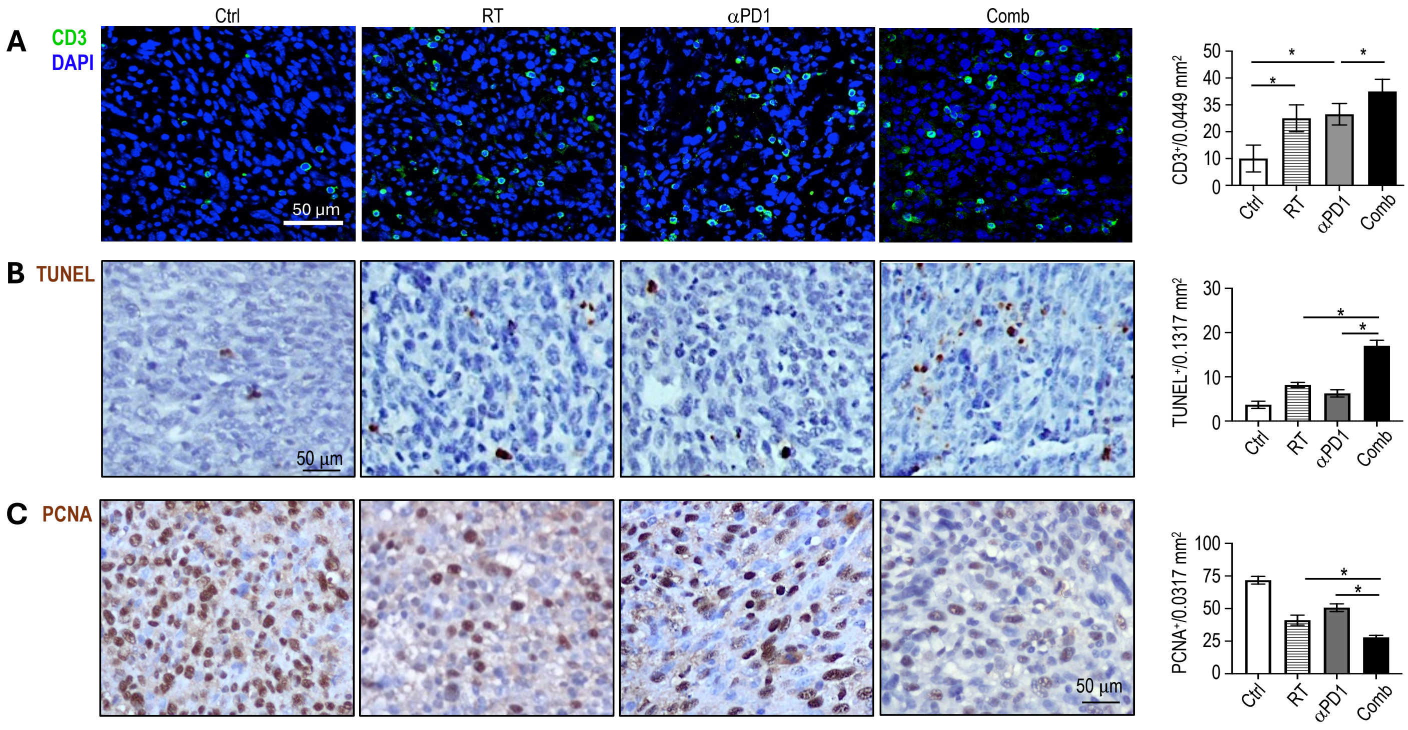

Figure S2. Combined radiotherapy and αPD1 treatment achieve enhanced efficacy in *Nf2*$^{-/-}$ model.

(A) Representative immunofluorescent staining images of CD3 in *Nf2*$^{-/-}$ tumors.
(B) Representative images of TUNEL staining for apoptotic cells
(C) Representative images of PCNA staining for proliferating tumor cells.

Image quantification and flow cytometry data are presented as mean ± s.d., and analyzed using Student's t-test and the Mann-Whitney test.

Table S1. Antibody panels.

Flow cytometry antibodies			
**Surface Markers**	**Clone**	**Fluorophore**	**Catalog Number**
CD11b	M1/70	APC-Cy7	BioLegend 101212
CD11c	N418	PE/Cy7	BioLegend 117324
CD206	C068C2	PE	BioLegend 141706
CD3	17A2	PE-cy7	BioLegend 100219
CD4	GK1.5	FITC	BioLegend 100405
CD45	30-F11	PerCP	BioLegend 103130
CD8	53-6.7	APC-cy7	BioLegend 100713
CD86	GL1	PE	BioLegend 105008
F4/80	BM8	PE-Cy7	BioLegend 123114
Foxp3	MF-14	PE	BioLegend 126403
Gr1	RB6-8C5	PE	BioLegend 108408
Granzyme B	QA16A02	APC	BioLegend 372204
IFN-γ	XMG1.2	PE/Dazzle594	BioLegend 505846
MHCII	M5/114.15.2	APC	BioLegend 107614
NK1.1	S17016D	APC	BioLegend 156505
TNF-α	MP6-XT22	PE	BioLegend 506306
**Treatment antibodies**			
**Target**	**Clone**	**Company**	**Catalog Number**
Anti-CD4	GK1.5	BioXCell	BE0003-1
Anti-CD8	2.43	BioXCell	BE0061
